# Contraceptive use among women who desire no more children in Indonesia: a cross-sectional study of a national survey

**DOI:** 10.51866/oa.519

**Published:** 2024-12-27

**Authors:** Maria Gayatri, Desy Nuri Fajarningtiyas

**Affiliations:** 1 MAPS, Research Center for Population, National Research and Innovation Agency, Jl. Gatot Subroto No. 10, South Jakarta, Indonesia. Email: desynuree@gmail.com; 2 MAPS, PhD, Directorate for Development of Service Quality of Family Planning, Ministry of Population and Family Development/National Population and Family Planning Board JI. Permata No. 1, East Jakarta, Indonesia.

**Keywords:** Fertility preferences, Contraceptive, Education, Women, Indonesia

## Abstract

**Introduction:**

The desire for no additional births may be used to estimate the demand for family planning. Couple education may influence contraceptive use. This study aimed to examine the relationship between education and contraceptive use among women who desire no more children in Indonesia.

**Methods:**

A dataset from the 2017 Indonesia Demographic and Health Survey (IDHS) was used. The sample consisted of 17,938 women aged 15-49 years who desired no more children. Binary logistic regression was conducted for data analyses.

**Results:**

Of the total respondents, 73% used contraceptive methods. Contraceptive use was strongly associated with secondary education among couples (odds ratio [OR] = 1.27; 95% confidence interval [CI] = 1.17–1.37) and the working status of the husband (OR=2.62; 95% CI=2.07-3.32). The respondents living in rural areas (OR=1.14; 95% CI= 1.06–1.23) and in the Java-Bali region (OR=1.36; 95% CI=1.27–1.46) were more likely to use contraceptive methods. However, the respondents aged more than 35 years were less likely to use contraceptives.

**Conclusion:**

Among women who desire no more children, contraceptive use is associated with educational level, age and place of residence. Educated women tend to be empowered and have better access to contraceptive services, improving their chances of using contraception. Healthcare providers and policymakers need to promote the use of contraceptive services including counselling for couples with a low educational level, urban women and women who live in the outer Java-Bali region.

## Introduction

The desire for no additional births may be used to estimate the demand for family planning. About 10.6% of married women aged 15–49 years in Indonesia who want to delay, space or limit their pregnancy do not use any contraceptive methods.^1^ The 2020–2024 National Medium-Term Development Plan in Indonesia targets to increase the contraceptive prevalence rate (CPR) to 63.41% and reduce the proportion of unmet needs to 7.4% by the end of 2024.^2^

Despite the achievement of the CPR target, which is 64% for the use of any contraceptive methods,^1^ reducing unmet needs seems challenging in Indonesia since the proportion has remained stagnant for a decade. The unmet need for family planning reflects the gap in fertility preference with contraceptive use.^3^ Contraceptive use remains crucial in Indonesia, as maternal mortality persists at a high rate of 189 per 100,000 live births.^4^ A previous study reported a 7% increase in the prevalence of contraceptive use, which may decrease maternal mortality by around 20% in Indonesia.^5^ The failure to meet the need for family planning contributes to an increase in unwanted pregnancies, which subsequently raises the risk of maternal mortality. Hence, studying contraceptive use, particularly among women who desire no more children and present a high risk for unwanted pregnancy, is noteworthy to understand the patterns of their contraceptive use.

Prior knowledge of contraceptive methods is needed to uptake contraception.^6^ Contraceptive continuation is determined by some factors including accurate and comprehensive counselling.^7,8^ The Method Information Index (MII), which is one of the indicators of counselling, is measured by (1) information on side effects and health issues related to the method used, (2) information on what to do if women experience side effects and (3) information on other contraceptive methods.^7,9^ According to the 2017 Indonesia Demographic and Health Survey (IDHS), only 29% of current users received information about all three MII aspects.^1^ A positive perception of family planning programmes is also influenced by the knowledge and desire to use contraceptives in the future.^10,11^ Moreover, knowledge, awareness and education are important factors in contraceptive use. Women with better education have better knowledge about their reproductive rights and responsibilities related to sexual and reproductive issues.^12^ Educated women also have better access to health information and services.^12^

Although many recent studies have evaluated contraceptive use in Indonesia, most of them have not specifically explored it relative to the intention to have more children.^13-15^ Given that 7.1% of both childbearing and pregnant women aged 15-49 years experience unwanted pregnancy,^1^ attention to women who no longer want to have another child is important. The contraceptive methods they use and to what extent socioeconomic factors including education contribute to their use must be assessed to create strategies for reducing unwanted pregnancy. Thus, the current study aimed to assess the relationship between education and contraceptive use among women who desire no more children in Indonesia. Specifically, this study investigated the use of both modern and traditional contraceptive methods relative to the educational backgrounds of women and their husbands. The study is expected to fill the research gap as well as provide some insights that may be useful for the development of family planning programmes targeting women who have no intention to have more children.

## Methods

**Figure 1:**
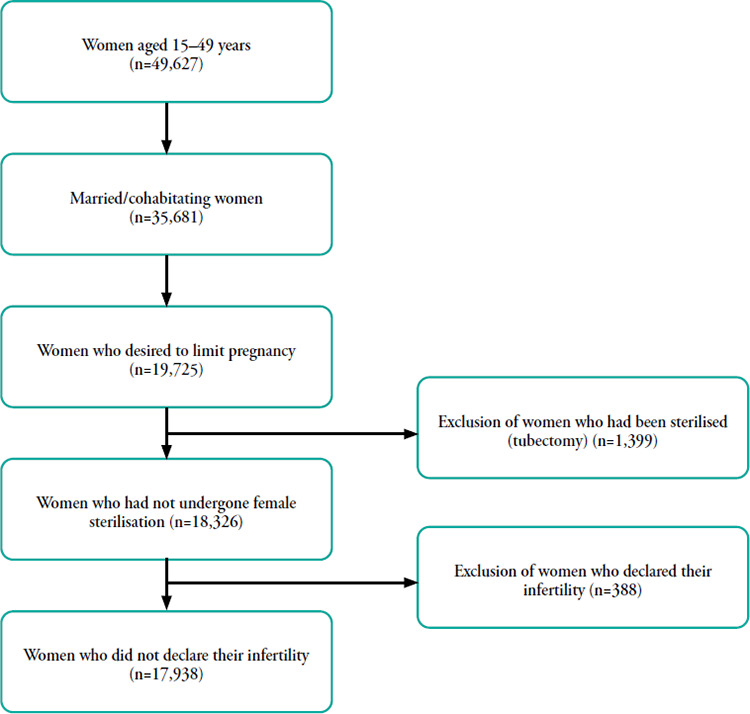
Respondent selection.

The dataset used in this study was obtained from the 2017 IDHS through the MEASURE DHS public repository website (www.dhsprogram.com).^1^ The IDHS is a nationally representative survey and is conducted in 34 provinces in Indonesia. The sample size of this study was 17,938 married or cohabitating women aged 15-49 years who desired to limit their pregnancy. Women who reported their infertility and had been sterilised (tubectomy) before the survey were excluded. The flowchart of respondent selection is presented in Figure 1.

For fertility intention, women were asked whether they wanted more children. If so, they were asked how long they would prefer to wait before their next pregnancies. The responses to the questions were then categorised as follows: ‘have another soon’, ‘have another later’, ‘have another, undecided when’, ‘undecided’, ‘want no more’, ‘sterilised’ and ‘declare infecund’. Since the dependent variable in this research was current contraceptive use among women who desired no more children, this research was limited to those who wanted no more children.

The dependent variable was dichotomous. ‘Users’ (coded as ‘1’) referred to women using any kind of modern or traditional birth control methods such as intrauterine devices (IUDs), implants, injectables, pills, male condoms, lactational amenorrhea methods, periodic abstinence, withdrawal, *jamu* (Indonesian traditional herbal drinks) and massages. ‘Non-users’ (coded as ‘0’) referred to women not using any contraceptive methods at the time of the survey.

Demographic and socioeconomic factors were included as the independent variables. The demographic variables included in the analysis were women’s age (15-34 vs 35-49 years) and the number of living children (0-2 vs ≥3). The socioeconomic variables included were women’s and their husbands’ educational level (primary, secondary vs higher), occupational status (working vs not working), place of residence (urban vs rural) and region (Java-Bali and outer Java-Bali). The classification of the region considered the population and economic circumstances that are considerably different between the Java-Bali region and the outer Java-Bali region. Additionally, the fertility rate in the Java-Bali region is mostly lower than that in other regions, which is a result of more health service-related infrastructures as well as skilled healthcare providers available in the region.^16^ Thus, the emphasis of family planning programmes also differs between the two regions.

Descriptive and inferential analyses were conducted on the weighted dataset using the weight variables provided in the survey. Descriptive statistics such as frequencies and percentages were applied, and a graph was developed to show the prevalence of contraceptive use among respondents. A bivariate analysis was performed using simple logistic regression and a multivariate analysis using multivariate binary logistic regression. Variables significant in the bivariate analysis were included in the multivariate analysis. The β-value was estimated using pseudo-maximum likelihood in the logistic regression model.^17^ Generally, this value measures the probabilities of a dependent variable for a certain predictor variable relative to the reference category of that variable. A crucial step before performing logistic regression is checking multicollinearity among variables. Multicollinearity occurs when independent variables are highly correlated, which can inflate the standard errors of regression coefficients. In this study, correlation matrices were used to check the correlation between the predictors, with a Spearman correlation coefficient of >0.8 indicating multicollinearity. Spearman correlation was used to check for multicollinearity due to the categorical nature of data.

The results of the simple bivariate logistic regression analysis were presented as unadjusted odds ratios, along with 95% confidence intervals (CIs), and the results of the multivariate logistic regression analysis as adjusted odds ratios (aORs) after adjusting for respondents’ age, number of living children, occupation, place of residence and region. STATA by StataCorp (College Station) version 15.1 was used to perform all analyses. For all associations, a P-value of <0.05 was considered statistically significant. The complex sample design was taken into consideration for the analysis of the IDHS dataset.

## Results

The sample comprised 17,938 women who desired no more children, and their mean age was 39.33 years (SD=6.47). The sociodemographic characteristics of the respondents are summarised in Table 1. More than three-fourths of the respondents (77%) were aged 35-49 years, while only 23% were aged 15-34 years. About 54% had zero to two living children, whereas only 46% had three or more living children. Regarding educational level, 43% of the respondents and 40% of their husbands completed primary education or lower; 48% of the respondents and 50% of their husbands, secondary education; and 9% of the respondents and 10% of their husbands, higher education. Most of the respondents (63%) and their husbands (63%) were employed. The proportion of the respondents living in rural areas was nearly similar to that of those living in urban areas. Conversely, 62% of the respondents lived in the Java-Bali region, while only 38% lived in the outer Java-Bali region.

**Table 1:** Characteristics of women who desired no more children according to the 2017 IDHS.

Characteristic	Women who desired no more children		
	Current non-user^*^ (n=4825)	Current user^*^ (n=13,112)	TOTAL^**^ (n=17,938)
Women’s educational level Primary or lower Secondary Higher	2,312 (29.8%) 2,100 (24.4%) 414 (26.2%)	5,452 (70.2%) 6,494 (75.6%) 1,166 (73.8%)	7,764 (43.3%) 8,594 (47.9%) 1,580 (8.8%)
Husbands’ educational level Primary or lower Secondary Higher	2,119 (29.3%) 2,267 (25.3%) 447 (25.7%)	5,121 (70.7%) 6,690 (74.7%) 1,294 (74.3%)	7,240 (40.4%) 8,957 (49.9%) 174 (9.7%)
Women’s age			
15-34 years	899 (21.7%)	3,239 (78.3%)	4,138 (23.1%)
35–49 years	3,927 (28.5%)	9,873 (71.5%)	13,800 (76.9%)
Number of living children			
0-2	2,474 (25.4%)	7,262 (74.6%)	9,736 (54.3%)
≥3	2,351 (28.7%)	5,851 (71.3%)	8,202 (45.7%)
Women’s working status			
Not working	1,786 (27.2%)	4,774 (72.8%)	6,560 (36.6%)
Working	3,039 (26.7%)	8,339 (73.3%)	11,378(63.4%)
Husbands’ working status			
Not working	141 (49.8%)	143 (50.2%)	284 (0.6%)
Working	4,684 (26.5%)	12,970 (73.5%)	17,654 (98.4%)
Place of residence			
Urban	2,372 (26.8%)	6,470 (73.2%)	8,842 (49.3%)
Rural	2,453 (27%)	6,643 (73%)	9,096 (50.7%)
Region			
Outer Java-Bali	2,021 (30%)	4,724 (70%)	6,745 (37.6%)
Java-Bali	2,804 (25%)	8,389 (75%)	11,193 (62.4%)
TOTAL	4,825 (26.9%)	13,113(73.1%)	17,938

The 2017 IDHS is the 2017 Indonesia Demographic and Health Survey

* Row total

** Column total

Approximately 73.1% of the respondents used contraceptive methods at the time of the survey. Although the differences were not quite significant, the proportion of current users was larger among those who were aged 15–34 years, had zero to two children, completed secondary education, were employed, and were living in the Java-Bali region. Conversely, in terms of the place of residence, the proportion of current users living in urban areas was almost the same as that of those living in rural areas.

**Figure 2:**
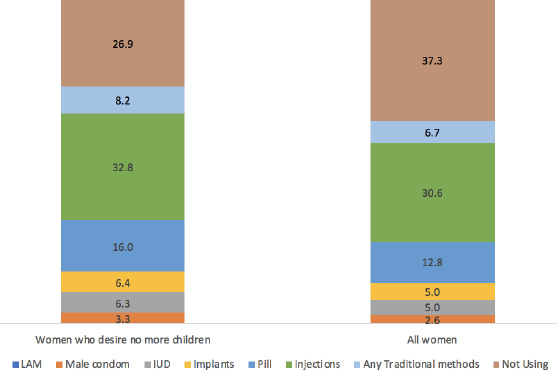
Prevalence of contraceptive use among women whodesired no more children according to the 2017 Indonesia Demographicand Health Survey (IDHS).

As illustrated in Figure 2, injectables (33%) and pills (16%) were the most favourable contraceptives reported by the respondents. The prevalence of IUDs and implants was nearly similar at 6% among the current users.

Table 2 summarises the results of the unadjusted and adjusted analyses between contraceptive use and respondent characteristics. The respondents who completed secondary education were more likely to use contraceptive methods (aOR=1.27; 95% CI= 1. 17–1.37) than those who completed primary education or lower. Moreover, the respondents whose husbands had secondary education were also more likely to use contraceptive methods (aOR=1.13; 95% CI=1.04–1.23) than those whose husbands had primary education or lower. The effect of educational level remained strong even after controlling for confounders such as age, parity, occupation, place of residence, and region.

**Table 2:** Unadjusted and adjusted analyses of the factors associated with contraceptive use among women who desired no more children according to the 2017 IDHS.

Covariate	Contraceptive use among women who desired no more children	
	Unadjusted OR (95% Cl)	aOR (95% Cl)
Women’s educational level Primary or lower Secondary Higher	1 1.23 (1.13-1.33)^*^ 1.09 (0.94-1.28)	1 1.27(1.17-1.37)^*^ 1.10(0.94-1.28)
Husbands’ educational level Primary or lower Secondary Higher	1 1.13 (1.04-1.23)^*^ 1.15 (0.99-1.34)	1 1.13(1.04-1.23)^*^ 1.15(0.99-1.34)
Women’s age 15–34 years 35–49 years	1 0.73 (0.67-0.79)^*^	1 0.72 (0.66-0.79)^*^
Number of living children 0-2 >3	1 0.97 (0.91-1.04)	- -
Women’s working status Not working Working	1 1.11 (1.04-1.19)^*^	1 1.11 (1.04-1.20)^*^
Husbands’ working status Not working Working	1 2.62 (2.07-3.32)^*^	1 2.62 (2.07-3.32)^*^
Place of residence Urban Rural	1 1.14(1.06-1.23)^*^	1 1.14(1.06-1.23)^*^
Region Outer Java-Bali Java-Bali	1 1.35 (1.26-1.45)^*^	1 1.36(1.27-1.46)^*^

The 2017 IDHS is the 2017 Indonesia Demographic and Health Survey

* Statistically significant at P<0.05

OR: odds ratio, aOR: adjusted odds ratio, CI: confidence interval

The respondents aged 35–49 years were less likely to use contraceptive methods than their counterparts (aOR=0.72; 95% CI=0.66–0.79). Regarding occupation, the respondents who were employed (aOR=1.11; 95% CI=1.04– 1.20) and respondents with employed husbands (aOR=2.62; 95% CI=2.07–3.32) were more likely to use contraceptive methods. The respondents living in rural areas (aOR=1.14; 95% CI=1.06–1.23) and in the Java-Bali region (aOR=1.36; 95% CI= 1.27–1.46) were more likely to be current users at the time of the survey.

## Discussion

The study examined couple education and contraceptive uptake among women who do not want to have any more children. According to the IDHS report, 53% of reproductive-age married women desired no more children, including those who had undergone tubectomy.^1^ Conversely, approximately 46% of married men aged 15–54 years desired no more children.^1^

This study showed that the CPR among the respondents was 73%. This rate is quite high compared to the prevalence reported in other developing countries, regardless of the methods used.^12,18^ The majority of the respondents still used short-acting contraceptive methods such as injectables and pills, which reflects a contradiction. In fertility limitation, the effectiveness of contraceptive methods in preventing pregnancy is crucial. Short-acting methods have higher discontinuation rates than long-acting and permanent methods because their effectiveness depends on users consistently and correctly following the prescribed usage.^1,19^

Among the respondents, 27% reported not using any contraceptive methods. Since this study focused on current contraceptive use, it is possible that some of those who reported not using any methods were past users who discontinued. Both discontinuation and the absence of contraceptive use are associated with unintended pregnancy,^20,21^ which may lead to unsafe abortions and devastating consequences on maternal and infant health.^22^ Fear of side effects is a common reason cited for not using as well as discontinuing the use of contraceptives.^23,24^ Thus, interventions aimed at increasing contraceptive use should address this concern. Improving comprehensive contraceptive knowledge and providing quality counselling are essential to address contraception-related issues, including side effects, to help reduce discontinuation and abstinence.

In the current study, the respondents who completed secondary education were more likely to use contraceptives to protect themselves from pregnancy than those who completed primary education or lower, which is in line with a previous report.^25^ Other studies have also reported the influences of maternal education on contraceptive use in women with no fertility intention.^26,27^ Educated women tend to have better knowledge and understanding of sexual and reproductive health and rights, including contraceptive use and behaviour.^28,29^ Moreover, they tend to be empowered by having equal roles in decision-making and health-seeking behaviour and have better access to contraceptive services, improving their chances of using contraception.^27,30^ Educated women are also more likely to understand and accept the importance of family planning and informed choice. They can use this information to decide which contraceptive methods are best suited to their reproductive health needs, including fertility intentions.^31,32^ Improving women’s education may play a significant role in increasing their knowledge and confidence to use contraceptives. Nevertheless, the impact of tertiary education on contraceptive use was not significant compared with that of primary education or lower in this study, which may be attributed to the small sample size of reproductive-age women who completed higher or tertiary education (<10% of the total sample).

Their husbands’ educational level also significantly influenced contraceptive use among the respondents. The study revealed that the respondents whose husbands completed primary education or lower were less likely to use contraceptive methods than those whose husbands completed secondary education. These results are similar to those of other studies.^26,28,30^ Better education among husbands and wives is more likely to yield comprehensive and accurate information and better discussion or communication on the benefits and utilisation of contraceptive methods.^28,30^ A lack of communication leads to women having incorrect assumptions regarding spousal preferences, including family size, which consequently discourages them from utilising family planning services.^33^ Therefore, the adoption of contraceptive methods is greater among educated couples.

In this study, maternal age was found to be significantly associated with contraceptive use among the respondents. The younger respondents were more likely to use contraceptive methods than the older respondents, consistent with the findings reported in another research.^26^ The findings may be explained by the following: (1) Younger women have longer reproductive periods; (2) younger women are more fertile than their counterparts, which increases their chances of getting pregnant; and (3) younger women are more likely to participate in the labour force and therefore prevent pregnancy by using contraceptives.

This study confirmed the effect of the employment status of both respondents and their husbands on contraceptive use. Contraceptive use among the working respondents as well as their working husbands was higher than that among their counterparts. The high prevalence of contraceptive use among working women is consistent with prior reports in Ethiopia,^12^ Zambia^26^ and Nigeria.^27^ A study conducted in India also showed that working women were more likely to desire no more children and use a contraceptive method than their counterparts.^34^ The possible reason is that working women may receive more information and education about reproductive health from their workplaces. They may also access contraceptive services in their company clinics or primary healthcare settings near their companies. Additionally, working is commonly related to financial ability. Working women may be less worried about healthcare expenditures, as they are more likely to afford contraceptive use-related costs by themselves.^18^ Moreover, pregnancy planning is essential for working women in terms of the cost and time they spend relative to childrearing, so using contraceptive methods is the best option for them.^32^ The relationship between employment status and contraceptive use is also attributed to women’s status, in which employed women are more likely to have equal power with their husbands in decision-making. Thus, they are actively involved in discussing their reproductive health, including determining the number of children and contraceptive methods they use. In many patriarchal societies, such as Indonesia, husbands are commonly the main decisionmakers in the household. Their role may be even stronger if women are unemployed, especially for financial considerations. Therefore, husbands’ disapproval of any contraceptive methods is one of the reasons for not using contraceptive methods. Furthermore, although a previous study found a non-significant relationship between husbands’ employment status and contraceptive use, which may be attributed to a greater influence of other sociodemographic factors,^35^ women with working husbands may benefit from their husbands’ higher awareness about their involvement in supporting family planning, such as breastfeeding practices. A study performed in Indonesia showed that working fathers were more likely to support and encourage their wives to practise breastfeeding.^36^ Exclusive breastfeeding is one of the non-hormonal contraceptive methods that effectively prevent pregnancy when applied correctly. Husbands’ involvement in joint childrearing, providing nutritional foods for their wives and offering emotional support is important in the success of the initiation and continuation of exclusive breastfeeding.^37^

The current study found that the respondents living in rural areas were more likely to use contraceptive methods than those living in urban areas. However, this result is inconsistent with other reports from Ethiopia and Bangladesh, in which urban women were more likely to use contraceptive methods because of better access to or close proximity to health facilities.^12,38^ Further, the 2017 IDHS showed that the predominant sources of family planning promotions were healthcare providers including midwives and family planning cadres or volunteers.^1^ Despite the wide implementation of family planning programmes within the country, implementing them is less difficult in rural areas in Indonesia. In addition, cadres or volunteers may be more active in rural areas than in urban areas, even if they earn a low salary. They show a strong commitment to their duties and work well in providing family planning-related information and services.^39^ Thus, it is possible that rural women are more likely to use contraception. This study also revealed a difference in the CPR among the respondents according to their region. The respondents living in the Java-Bali region were more likely to use contraceptive methods than those living in the outer Java-Bali region. Another study used the same 2017 IDHS dataset and reported a similar result.^14^

Community-based education and mass media campaigns need to be promoted to improve contraceptive use in Indonesia. Conducting workshops or capacity-building programmes at the village or community level that involve local healthcare workers or peer groups is important to raise awareness about available contraceptive choices, especially long-acting reversible contraceptives (LARCs). Mass media campaigns via television and social media platforms are crucial to disseminating accurate and comprehensive information about contraception. Such campaigns are important to address common misconceptions about contraception by collaborating with religious leaders, influencers or public figures to promote family planning adoption.

Extending the accessibility of LARCs can be increased by offering free contraceptive services under national health assurance programmes and conducting massive family planning services, especially in remote and rural areas in Indonesia. Strengthening the quality of counselling and family planning services is also important, including follow-up services to address any side effects or complications due to contraceptive adoption. Furthermore, promoting male involvement is needed to inform men about the benefits of contraception and encourage them to share decision-making in contraceptive use with their wives.

The strength of this study is the national coverage of the survey with a large sample size. Moreover, the IDHS collects comprehensive information on demographic and health-related variables using standardised questionnaires, which are also applied in 89 other countries, ranging from countries in Sub-Saharan Africa to countries in Latin America and the Caribbean region.^40^ However, the study also has limitations. The data were based on the respondents’ self-reports at the time of the survey, which may lead to response bias. Self-reports may indicate what the respondents wanted at the time of the survey but not necessarily show their actual practices and behaviours in their sexual relationships. Moreover, this study considered women’s points of view but did not include the fertility preferences of their husbands. Due to the crosssectional design, cause-and-effect relationships could not be established in this study.

In conclusion, couple education plays a significant role in contraceptive use among women who desire no more children. Healthcare providers and policymakers need to consider promoting contraceptive services including counselling for couples with a low educational level, urban women and women living in the outer Java-Bali region. It is recommended to improve Information, Education, and Communication (IEC) and counselling among couples who desire no more children, especially in the workplace. Further studies can be conducted by considering both husbands’ and wives’ fertility desires and cultural norms.

## Data sharing statement

Data would be made available from the corresponding author upon a reasonable request.

**How does this paper make a difference in general practice?**

- The study does not provide specific data regarding the demand for family planning. However, it presents information about the prevalence of contraceptive use among women who desire no more children, which healthcare providers and general practitioners can use to predict family planning practices and offer quality contraceptive choices.
- The findings offer information about targeted users of contraceptive methods, which can inform the delivery of the most suitable services.
- The findings can guide healthcare providers and general practitioners in educating clients to choose a suitable contraceptive method based on their fertility goals and health concerns.
